# Supplementary material for: Modeling the emergence of viral resistance for SARS-CoV-2 during treatment with an anti-spike monoclonal antibody
Source: PLoS Pathog. 2024 Apr 18;20(4):e1011680. doi: 10.1371/journal.ppat.1011680 (PMC11060554; doi:10.1371/journal.ppat.1011680)
Supplement: S1 Text — Table A. Antibody concentration exponential decay rate estimated from Amax and A28, where A28 is the antibody concentration at day 28. B2-7 does not have information on Amax, so the average Amax for the other 6 participants is used instead. The values for α and Amax are used in Eq (4). Table B. Fitting comparison using different estimates of γ relative to c. Table C. Definition, units and values of model parameters. Note that the subscripts 1, 2 for each parameter refers to the sensitive and resistant virus, respectively. If a fitted parameter is shared between the two models, the population estimate for the logistic proliferation model is reported first, followed by the innate immune response model. Values with (*) is the average of the estimated values for the seven participants. The values for kon reported in Table S3 are in unit of M-1 s-1, while the computation is done using a conversion to mL μg-1day-1 (see main text). Fig A. Fit of the target cell limited model with drug sensitive and resistant viral strain to the data. The circles represent viral load data, where filled green and red circles indicate viral populations dominated by either the sensitive or resistant viral strain, respectively. The unfilled circles are data below the limit of quantification or limit of detection (indicated by the two horizontal lines). Black curves are the best-fit of the model to the total viral load. The dashed–green or–red curves are model simulation of the sensitive or resistant viral strain, respectively. The first vertical red (dashed-dotted) line indicates the timing of treatment. The second vertical red (dashed) line indicates the estimated time when adaptive immunity begins to emerge. The dashed black curve shows the total viral load without treatment. Fig B. Long-term viral load in the logistic and innate immune response models without adaptive immunity. Simulations are done using the best fit parameters of participant B2-8 for both models. Fig C. Innate immune respon [file ppat.1011680.s001.docx]

**Supplementary Material**

Modeling the emergence of viral resistance in SARS-CoV-2 patients treated with an anti-spike monoclonal antibody

**Section A. Resistant mutations are likely to exist at baseline**

Following Rong et al. [1], we determine the likelihood that any single mutation exists at the time of treatment. Let $L$ denote the SARS-CoV-2 genome length in nucleotides and $\mu$ be the probability that RNA polymerase miscopies a base. Then the probability of having i-substitutions in one replication event is:

$$P_{i}=\binom{L}{i}\mu^{i}\left( 1-\mu\right)^{L-i} ,$$

for large $L$ and small $\mu$ this is well approximated by a Poisson distribution with mean $\lambda=L\mu$, where $L\mu$ is the expected number of miscopied bases in the genome. The SARS-CoV-2 genome is about 3 x10^4^ nucleotides long and the mutation rate for SARS-CoV-2 is ~ ${10}^{-6}$ per site per replication cycle [2,3]. Thus, we expect $\lambda=0.03$ mutations per genome every time a virion is produced.

Because the viral clearance rate$c$ is much larger than the death rate of infected cells we expect the viral load$V$ and the number of infected cells$I$ to quickly reach a quasi-steady state in which the total viral production rate $P_{T}=\pi I=cV$ [4]. We assumed $c=10$/day based on prior estimates, so after the first day or so of infection $P_{T}=10 V(t)$, where according to our logistic proliferation and innate immune response models $V(t)$ reaches a peak of ~ $5\times{10}^{8}$ RNA copies/ml or more for the individuals under study prior to the start of treatment (Figs. 1 and 2). The volume of the upper respiratory tract is about 30 ml [5]. Thus, the total daily viral production at the viral peak is at least $P_{T}=10\times5\times30\times{10}^{8}=1.5\times{10}^{11}$ copies/day. Alternatively, one may assume that the viral load measured from 5-20 swabs would be representative of the total number of viruses in the upper respiratory tract, giving estimates of $P_{T}$ between $2.5\times{10}^{10}$ to $1.0\times{10}^{11}$ copies/day.

As we previously showed for influenza infection [6], the total area under the clearance rate, c, times the viral load curve, *V*(*t*), for an acute infection can be approximated by summing the contributions from one day before to one day after the viral peak [6] and thus will be slightly larger than the total peak viral production in the upper respiratory tract, say ~$2\times{10}^{11}$ copies (One can also formally calculate the area under c times the viral load curve but for our purposes here a rough estimate will do). As each base in the genome can mutate to 3 others the total number of possible sequences with 1-base changes is$9\times{10}^{4}$. With over 10^11^ viruses generated by the peak, each possible 1-base mutation will be made millions of times before BAM treatment and if sufficiently fit should pre-exist when treatment is initiated. The most prevalent drug resistance mutations were E484K and E484Q, both of which require only a single base change to generate (a G to A for E484K and a G to C for E484Q). This implies that even in an infection by 100% wild type virus we expect by the time of symptom onset and treatment initiation these mutations should pre-exist, especially since the E484K mutant has a higher affinity for the ACE2 receptor than the wild type virus [7].

**Section B. Pharmacokinetic information for the seven patients**

The maximum concentration of antibody measured after the infusion and at day 28 (last day of the trial) are known [8]. Thus, we estimated each individual’s BAM pharmacokinetic parameters and used them in our fitting. We used a simple exponential decay model to fit the two available data points (antibody concentration at max and at day 28) to estimate and decay rate. Then, we used the decay rate and the maximum concentration information for the fitting of each person (see Table A).

|  | B2-2 | B2-3 | B2-4 | B2-5 | B2-6 | B2-7 | B2-8 |
| --- | --- | --- | --- | --- | --- | --- | --- |
| $\boldsymbol{A}_{\boldsymbol{max}}$  ($\boldsymbol{\mu g/mL}\boldsymbol{)}$ | 261.13 | 243.46 | 334.22 | 228.21 | 164.21 | 235.83* | 143.92 |
| $\boldsymbol{A}_{\boldsymbol{28}}$  $\boldsymbol{(\mu g/mL}\boldsymbol{)}$ | 15.74 | 35.91 | 37.51 | 37.02 | 2.54* | 37.12 | 21.41 |
| $\boldsymbol{\alpha}$  (1/day) | 0.100 | 0.068 | 0.078 | 0.065 | 0.149 | 0.066 | 0.068 |

**Table A.** Antibody concentration exponential decay rate estimated from $A_{max}$ and$A_{28}$, where $A_{28}$ is the antibody concentration at day 28. B2-7 does not have information on$A_{max}$, so the average $A_{max}$ for the other 6 participants is used instead. The values for $\alpha$ and $A_{max}$ are used in Equation (4).

**Section C. Target cells are likely limited at the time of treatment**

At first glance, a reasonable explanation for the emergence of resistant virus associated with viral rebound of 3-4 log is that the resistant viral strains are more fit than the wild-type virus in the presence of BAM. However, the probability that a resistant strain such as E484K is present at the time of treatment is very likely (see Section A) and yet only 8 out of 111 treated patients had detectable resistant virus. Together, these observations suggest that the presence of a resistant mutant serves as a necessary condition for the transient viral rebound but is not sufficient to guarantee the emergence of the mutant strain or lead to the transient viral rebound.

To examine the situation objectively, we consider the classical target cell limited model:

$$T^{'}=-\beta VT,$$

$$I^{'}=\beta VT-\delta I,$$

$$V^{'}=pI-cV.$$

The reproduction number is$R=\frac{\beta pT}{\delta c}$, where the basic reproduction number is$R_{0}=\frac{\beta pT(0)}{\delta c}$. Without treatment, *R* is greater than 1 until the viral peak is reached and then decreases as target cells are eliminated, which is why viral load also declines. Moreover, because resistance mutants were not observed in the absence of BAM, it is expected that the resistant has a reproduction number (*R*_R_) lower than that of the sensitive (*R*_S_). As symptom onset usually occurs near the viral peak and BAM treatment was started after symptom onset and >5 days after symptom onset in the majority of study participants [8] the viral load is expected to be declining at the time of treatment [9,10]. At this time, *R*_S_<1 and for the resistant mutant to increase, it needs to have *R*_R_>1, implying that BAM treatment is able to increase *R*_R_ from <1 to larger than 1. This can only occur if BAM generates antibody dependent enhancement of infection, which has not been see in vivo (see discussion in the main text), or if it leads to an increase in target cells, which we explore in this study. Without the latter, it is hard to explain the increase in the resistant mutant. And indeed, the classical target cell limited model shown above does not produce the rebound with resistant virus when treatment is started in the post-peak phase of the viral dynamics. We tested variations of the standard target cell limited model. One such best-fits using the target cell limited model (Eqs. 1-5, main text) shows that viral load has yet to peak at the time of treatment (dashed black curves, Fig A), which contradicts our expectation.


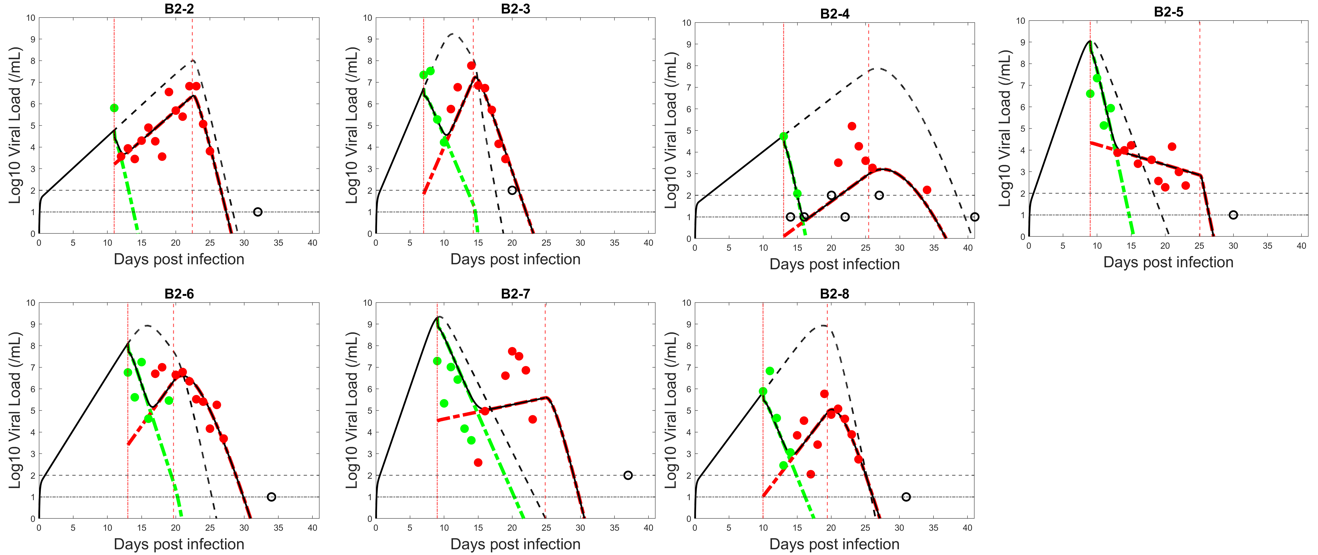
**Fig A.** Fit of the target cell limited model with drug sensitive and resistant viral strain to the data. The circles represent viral load data, where filled green and red circles indicate viral populations dominated by either the sensitive or resistant viral strain, respectively. The unfilled circles are data below the limit of quantification or limit of detection (indicated by the two horizontal lines). Black curves are the best-fit of the model to the total viral load. The dashed–green or –red curves are model simulation of the sensitive or resistant viral strain, respectively. The first vertical red (dashed-dotted) line indicates the timing of treatment. The second vertical red (dashed) line indicates the estimated time when adaptive immunity begins to emerge. The dashed black curve shows the total viral load without treatment.

**Section D. Testing the best values for** $\boldsymbol{\gamma}$

Setting the association constant $k_{on}$ to $5.5\times{10}^{5}M^{-1}s^{-1}$ and dissociation constant $k_{off}$ to$2.5\times{10}^{-5} s^{-1}$, we test possible values of$\gamma$, the clearance rate of antibody-virion complexes. We find that small variations in$\gamma$ do not result in significant changes to the model goodness of fit (Table B). For our final models, we set$\gamma=3c$.

| $\gamma$ | Logistic proliferation model (-2LL) | Innate immune response model (-2LL) |
| --- | --- | --- |
| $1c$ | $191.49$ | $190.57$ |
| $2c$ | $190.19$ | $185.27$ |
| $3c$ | $191.59$ | $190.68$ |
| $4c$ | $189.79$ | $186.82$ |
| $5c$ | $190.64$ | $188.67$ |

**Table B.** Fitting comparison using different estimates of $\gamma$ relative to$c$.

**Section E. Summary of parameter definitions and units**

We summarize the definition, units and values of model parameters in Table C.

| Parameter | Definition & Units | Value | Reference |
| --- | --- | --- | --- |
| $\beta$ | Infection rate $\left( mL RNA copy^{-1}\mathrm{da}y^{-1} \right)$ | ${10}^{-8.88}$ /$10^{-8.48}$ | Fitted |
| $\delta$ | Baseline infected cell death rate $\left( \mathrm{da}y^{-1} \right)$ | $2.28$ /$2.38$ | Fitted |
| $k$ | Progression rate from the eclipse phase to productive infection $\left( \mathrm{da}y^{-1} \right)$ | 4 | [4] |
| $\pi$ | Composite parameter for virus production and sampling $\left( copies mL^{-1}\mathrm{da}y^{-1} \right)$ | $1175.5$ / $1102.28$ | Fitted |
| $c$ | Virus clearance rate $\left( \mathrm{da}y^{-1} \right)$ | 10 | [4] |
| $\phi$ | Conversion rate from target to refractory cells $\left( \mathrm{cell}s^{-1}\mathrm{da}y^{-1} \right)$ | $7.9\times{10}^{-7}$ | Fitted |
| $\rho$ | Reversion rate from refractory to target cells $\left( \mathrm{da}y^{-1} \right)$ | $5.30\times{10}^{-2}$ | Fitted |
| $r$ | Maximum proliferation rate of target cells $\left( \mathrm{da}y^{-1} \right)$ | $0.79$ | Fitted |
| $T_{M}$ | Homeostatic size of the target cell population $(cells)$ | $8\times{10}^{7}$ | [4] |
| $\sigma$ | Adaptive immune response emergence rate $\left( \mathrm{da}y^{-1} \right)$ | $0.27$ / $0.24$ | Fitted |
| $t^{*}$ | Time when adaptive immune response begins to emerge $\left( \mathrm{days} \right)$ | $15.75$ / 18.18 | Fitted |
| $t^{**}$ | Time when resistant viruses emerge and grow $(days)$ | ${3.67}^{(*)}$ / ${2.33}^{(*)}$ | Estimated |
| $\delta_{M}$ | Maximum infected cell clearance rate due to the adaptive immune response $\left( \mathrm{da}y^{-1} \right)$ | $10\delta$ | Assumed |
| $\mu$ | Mutation rate $\left( per base copied \right)$ | ${10}^{-6}$ | [2,3] |
| $\gamma$ | Virus-mAb immune complex clearance rate $\left( \mathrm{da}y^{-1} \right)$ | $3c$ | See Section D |
| $k_{on}$ | Formation rate of immune complexes $\left( mL \mu g^{-1}\mathrm{da}y^{-1} \right)$ or $\left( M^{-1}s^{-1} \right)$ | $5.5\times{10}^{5}$ | [11] |
| $k_{off}$ | Dissociation rate of immune complex $\left( s^{-1} \right)$ | $2.5\times{10}^{-5}$ | [11] |
| $A_{max}$ | Maximum serum concentration of BAM $\left( \mu g mL^{-1} \right)$ or $\left( \mathrm{mol}L^{-1} \right)$ |  | See Section B |
| $\alpha$ | Clearance rate of BAM $\left( \mathrm{da}y^{-1} \right)$ |  | See Section B |
| $\Delta_{T}$ | Infusion duration $\left( \mathrm{day} \right)$ | $1/24$ | Assumed |

**Table C.** Definition, units and values of model parameters. Note that the subscripts 1, 2 for each parameter refers to the sensitive and resistant virus, respectively. If a fitted parameter is shared between the two models, the population estimate for the logistic proliferation model is reported first, followed by the innate immune response model. Values with${}^{(*)}$is the average of the estimated values for the seven participants. The values for$k_{\mathrm{on}}$ reported in Table C are in unit of$M^{-1}s^{-1}$, while the computation is done using a conversion to$mL \mu g^{-1}\mathrm{da}y^{-1}$ (see main text).

**Section F. Immune response and logistic proliferation models without adaptive immunity**

Adaptive immunity plays an important role in the ultimate clearance of virus. To demonstrate this effect, we use the best fit parameters for B2-8 from both models to simulate what would happen without adaptive immunity (and without treatment, which does not affect the conclusion). In both cases, we find that the viral load is sustained at high level for a long time (Fig B).


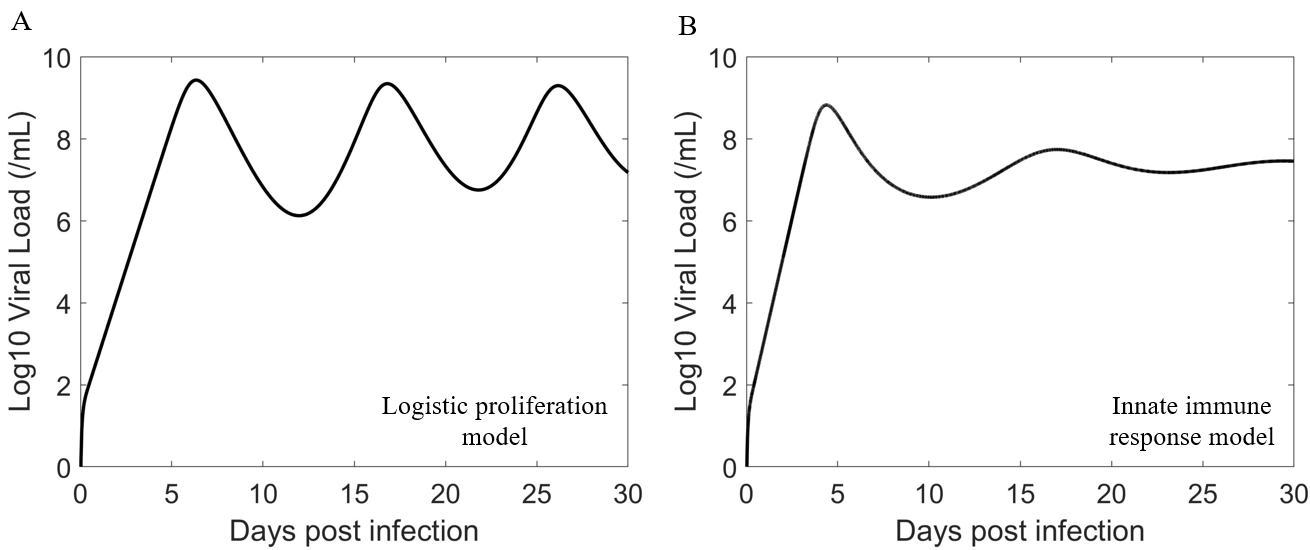
**Fig B.** Long-term viral load in the logistic and innate immune response models without adaptive immunity. Simulations are done using the best fit parameters of participant B2-8 for both models.

**Section G.** $\boldsymbol{\rho}$ **and** $\boldsymbol{\phi}$ **comparison between rebound and non-rebound individuals in the cohort that received treatment with 700 mg BAM**

We fit the innate immune response model to all treated individuals who received 700 mg BAM and did not rebound. Since we do not have the individual’s antibody level, we used the concentration provided by the FDA (196 mg/$\mu$L) and a drug washout rate $\alpha=0.0394$ per day. Additionally, there is no resistance for this group of individuals, so we only fit to the total viral load. The model and the fitting are otherwise identical.

We then compared the distributions of the innate immune response parameters $\rho$ and $\phi$ between those who rebound (n=7 in the main text) and those who did not rebound (n=102) (Fig H). Specifically, while the values of $\phi$ for rebound individuals are slightly lower than non-rebound, the values of $\rho$ for rebound individual are about at least one order of magnitude higher than non-rebound. We provided the best fit in Figs C-D. For completeness, we also carried out the comparison using the data for the placebo group and found a similar result for ρ and φ (Figs E-G, and I).


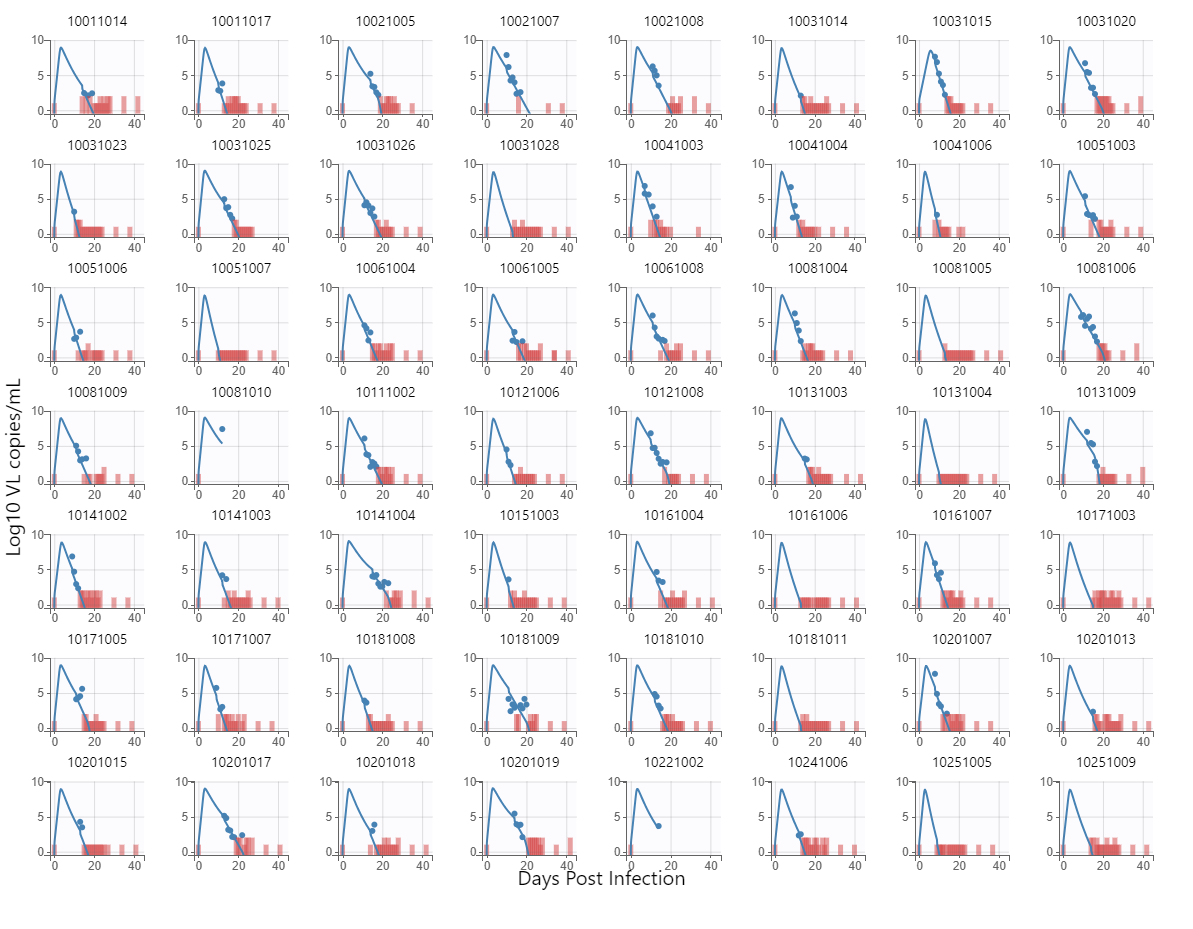


**Fig C.** Innate immune response model fit to data from patients who were treated with 700 mg BAM and did not rebound (part 1). The red bars indicate the limit of quantification (higher bar) and the limit of detection (lower bar).


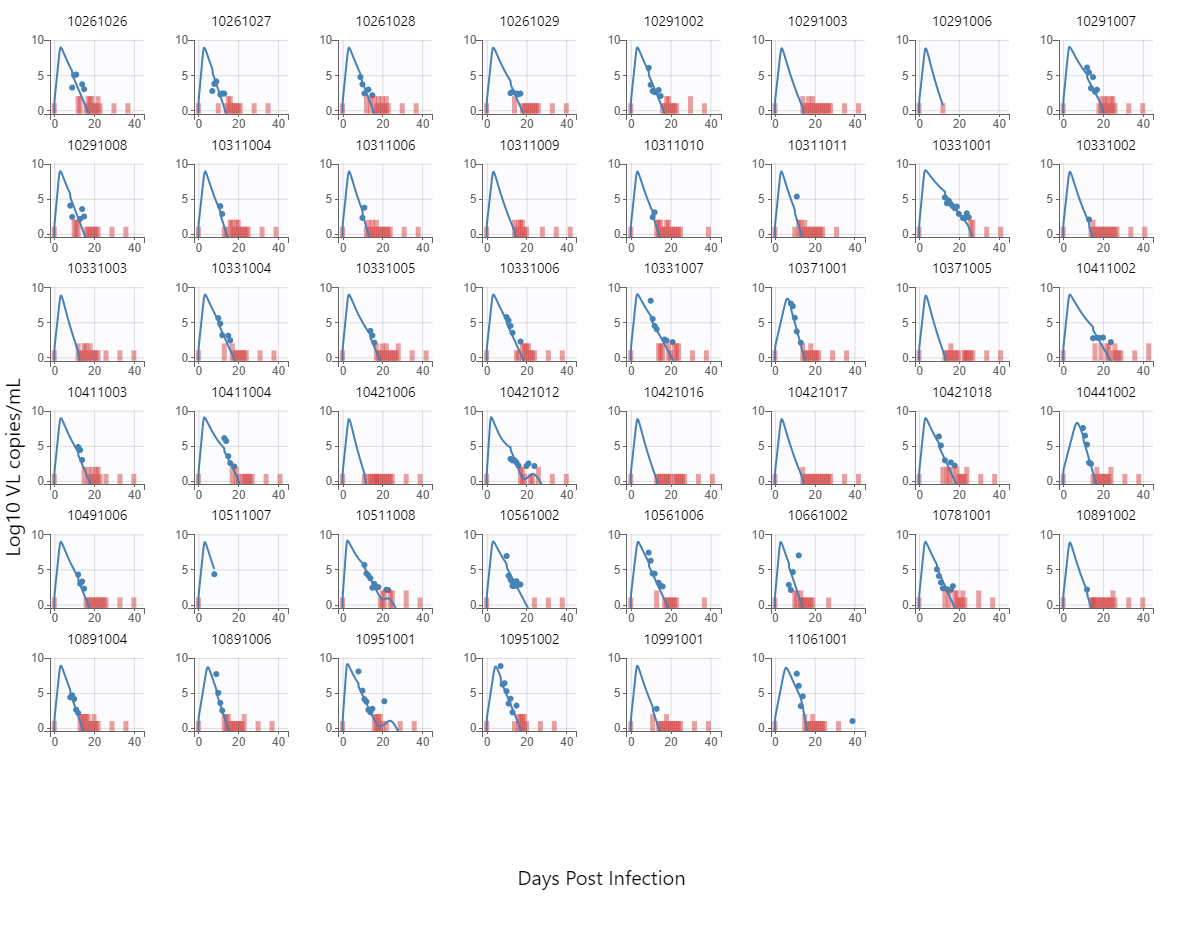


**Fig D.** Innate immune response model fit to data from patients who were treated with 700 mg BAM and did not rebound (part 2). The red bars indicate the limit of quantification (higher bar) and the limit of detection (lower bar).


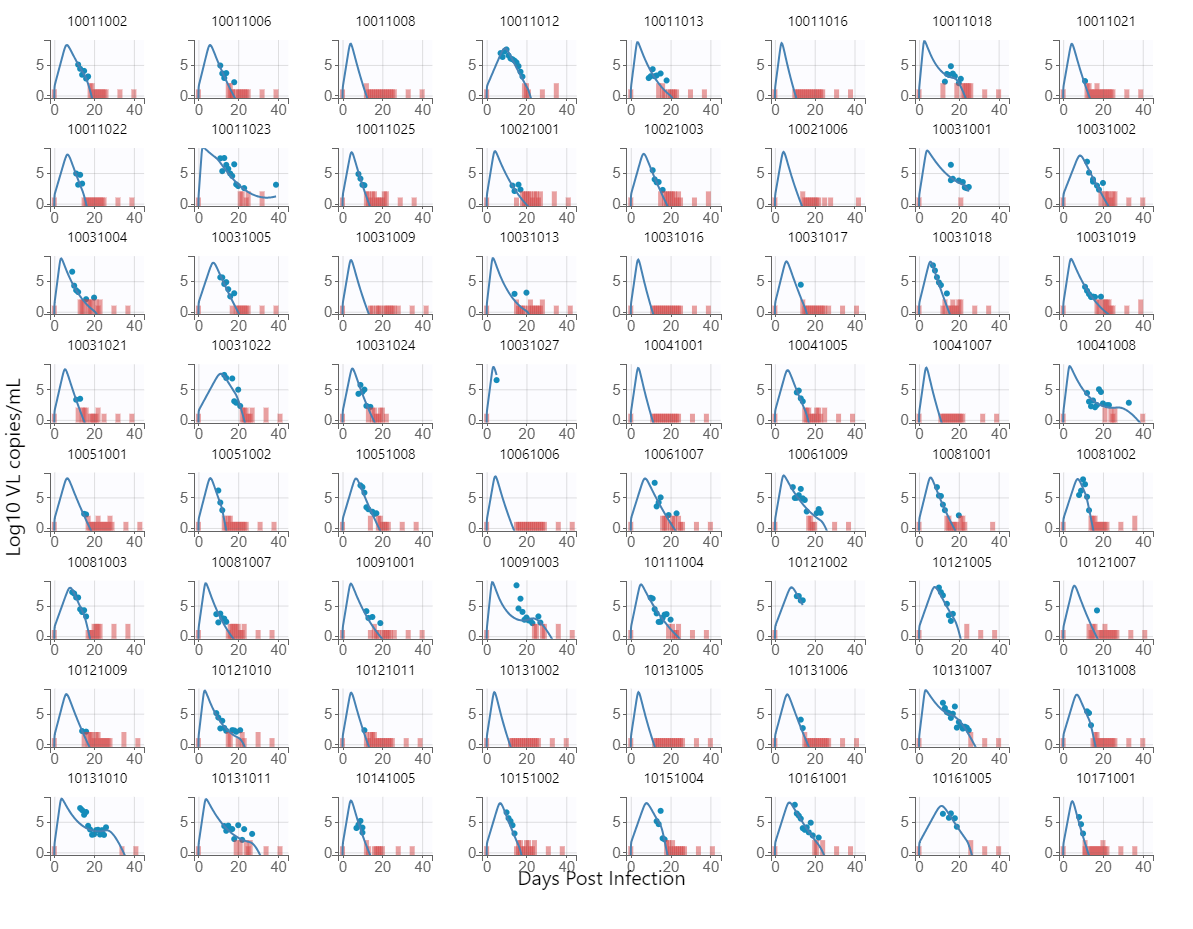


**Fig E.** Innate immune response model fit to data from control patients (part 1). The red bars indicate the limit of quantification (higher bar) and the limit of detection (lower bar).


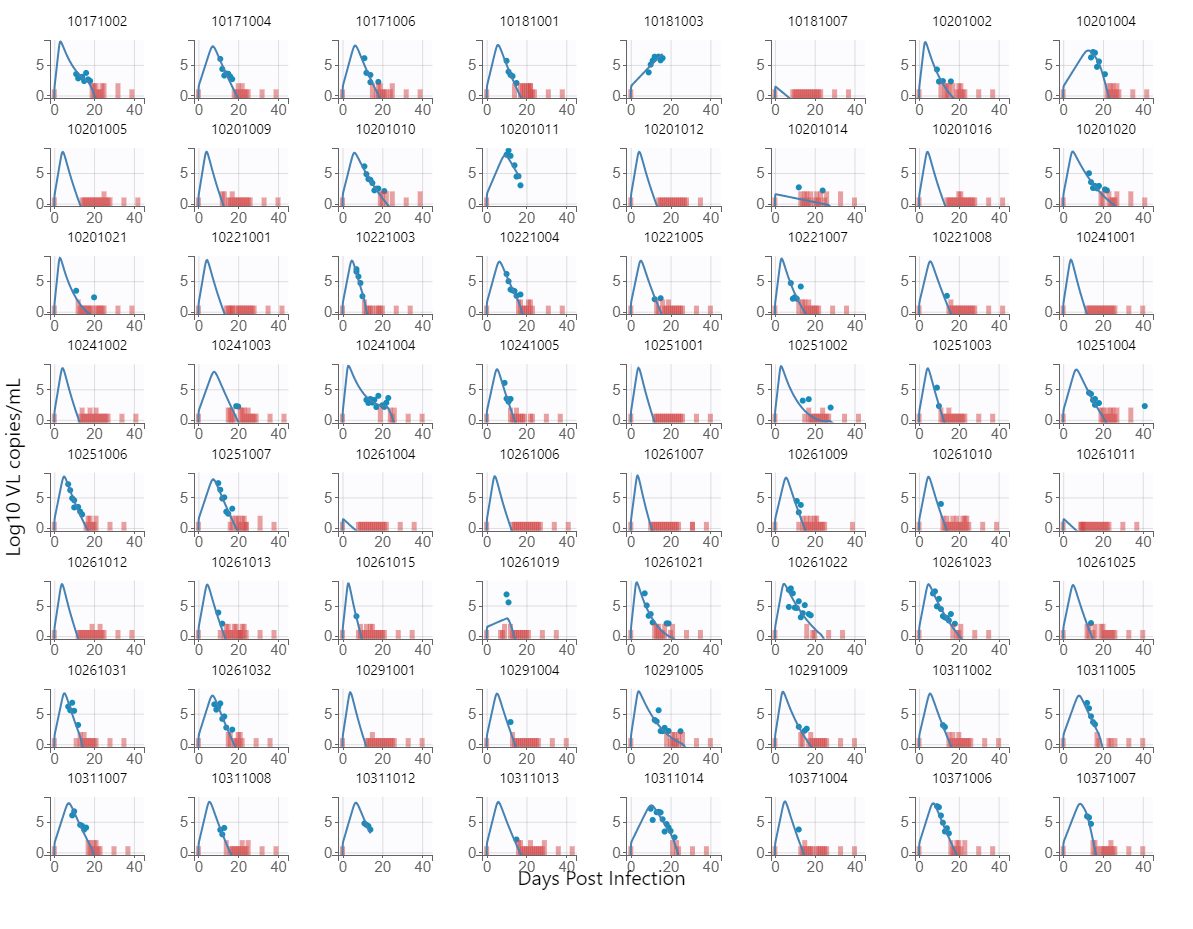


**Fig F.** Innate immune response model fit to data from control patients (part 2). The red bars indicate the limit of quantification (higher bar) and the limit of detection (lower bar).


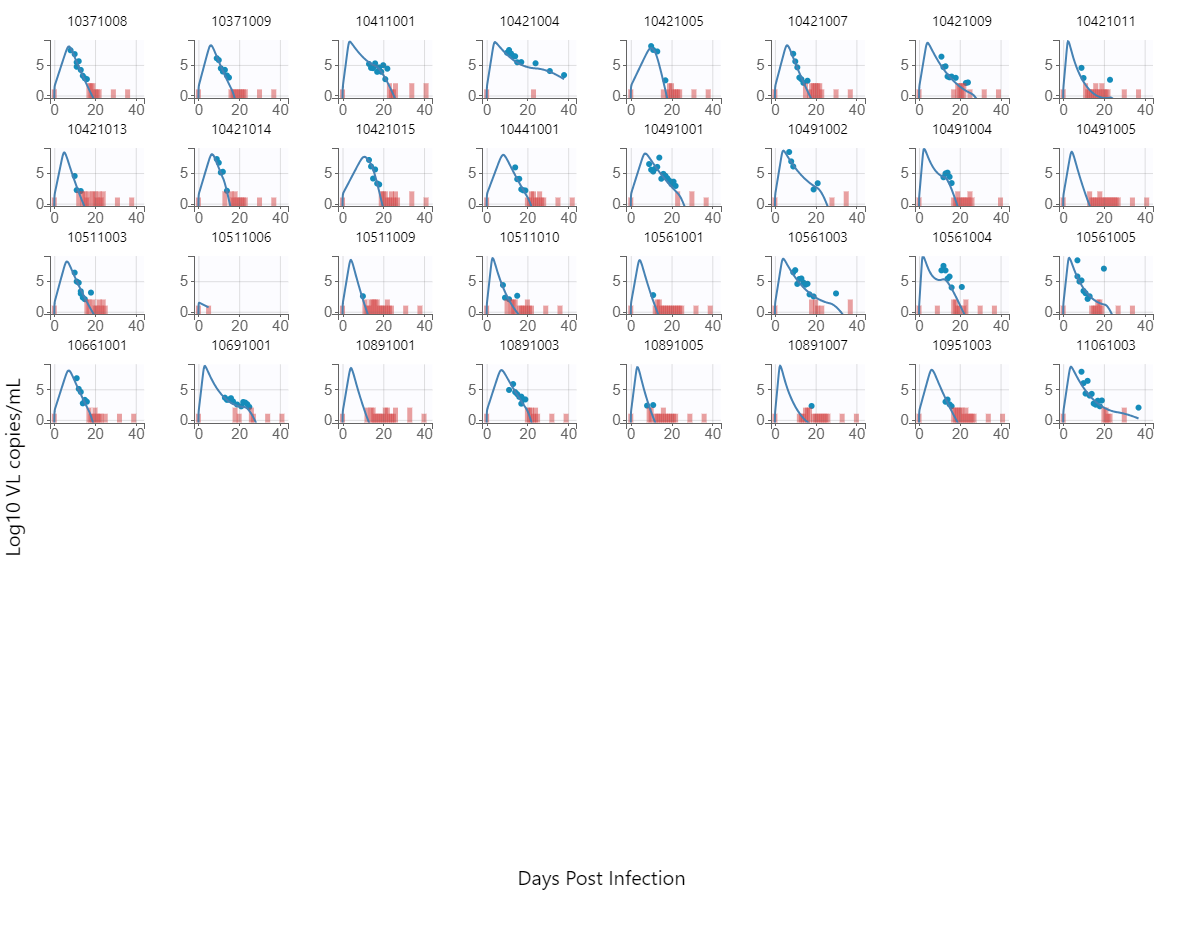


**Fig G.** Innate immune response model fit to data from control patients (part 3). The red bars indicate the limit of quantification (higher bar) and the limit of detection (lower bar).


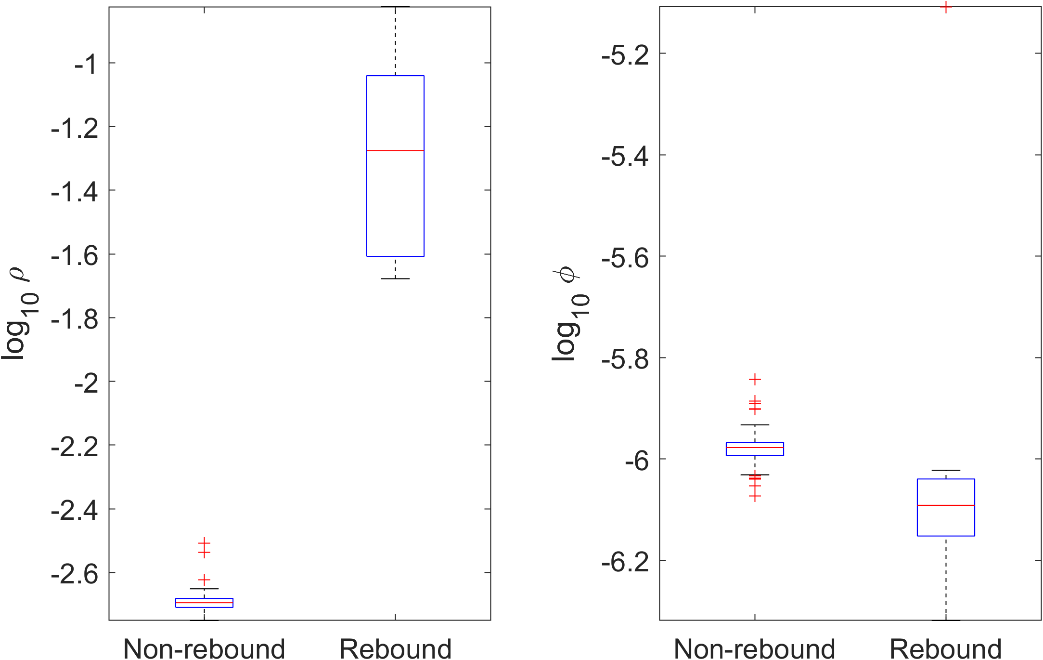


**Fig H.** Distributions of $\rho$ and $\phi$ for rebound (n=7) and non-rebound (n=102) treated individuals with 700 mg BAM. Boxes in boxplots start in the first quartile and end in the third quartile of the data. The line is the median and the whiskers connect the top/bottom of the box to the max/min values that are not an outliers (data points further than 1.5 $\times$ IQR). Red crosses are outliers (outside of the whisker range).


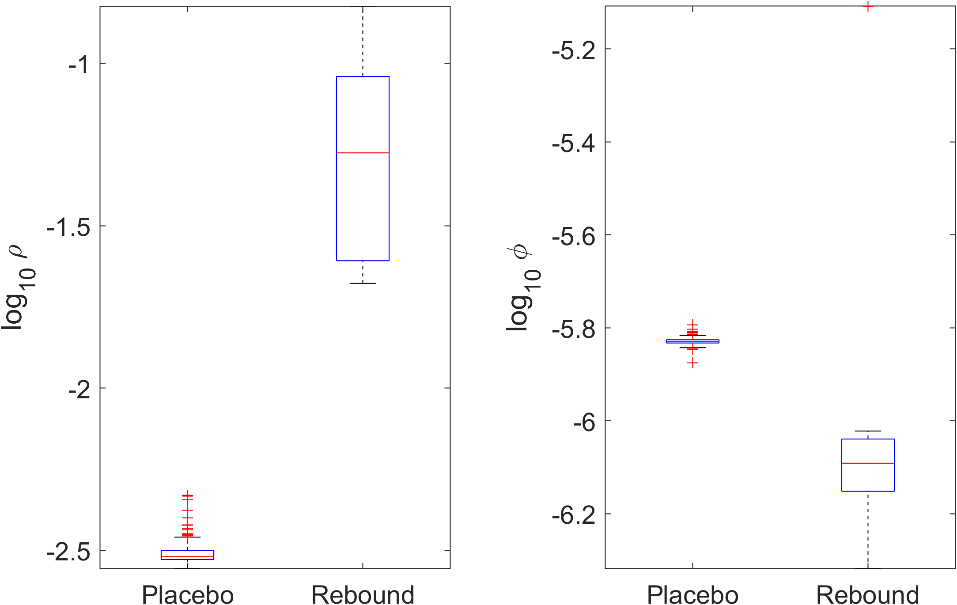


**Fig I.** Distributions of $\rho$ and $\phi$ for treated (700 mg BAM) rebound (n=7) and placebo (n=160) individuals. Boxes in boxplots start in the first quartile and end in the third quartile of the data. The line is the median and the whiskers connect the top/bottom of the box to the max/min values that are not an outliers (data points further than 1.5 $\times$ IQR). Red crosses are outliers (outside of the whisker range).

**References**

1. Rong L, Dahari H, Ribeiro RM, Perelson AS. Rapid Emergence of Protease Inhibitor Resistance in Hepatitis C Virus. Sci Transl Med. 2010 May 5;2(30). Available from: https://www.science.org/doi/10.1126/scitranslmed.3000544

2. Amicone M, Borges V, Alves MJ, Isidro J, Zé-Zé L, Duarte S, et al. Mutation rate of SARS-CoV-2 and emergence of mutators during experimental evolution. Evol Med Public Health. 2022 Jan 5;10(1):142–55.

3. Bar-On YM, Flamholz A, Phillips R, Milo R. SARS-CoV-2 (COVID-19) by the numbers. eLife. 2020 Apr 2;9:e57309.

4. Ke R, Zitzmann C, Ho DD, Ribeiro RM, Perelson AS. In vivo kinetics of SARS-CoV-2 infection and its relationship with a person’s infectiousness. Proc Natl Acad Sci. 2021 Dec 7;118(49):e2111477118.

5. Ménache MG, Hanna LM, Gross EA, Lou SR, Zinreich SJ, Leopold DA, et al. Upper respiratory tract surface areas and volumes of laboratory animals and humans: considerations for dosimetry models. J Toxicol Environ Health. 1997 Apr;50(5):475–506.

6. Perelson AS, Rong L, Hayden FG. Combination Antiviral Therapy for Influenza: Predictions From Modeling of Human Infections. J Infect Dis. 2012 Jun 1;205(11):1642–5.

7. Barton MI, MacGowan SA, Kutuzov MA, Dushek O, Barton GJ, Van Der Merwe PA. Effects of common mutations in the SARS-CoV-2 Spike RBD and its ligand, the human ACE2 receptor on binding affinity and kinetics. eLife. 2021 Aug 26;10:e70658.

8. Chew KW, Moser C, Daar ES, Wohl DA, Li JZ, Coombs RW, et al. Antiviral and clinical activity of bamlanivimab in a randomized trial of non-hospitalized adults with COVID-19. Nat Commun. 2022 Aug 22;13(1):4931.

9. Wölfel R, Corman VM, Guggemos W, Seilmaier M, Zange S, Müller MA, et al. Virological assessment of hospitalized patients with COVID-2019. Nature. 2020 May 28;581(7809):465–9.

10. Killingley B, Mann AJ, Kalinova M, Boyers A, Goonawardane N, Zhou J, et al. Safety, tolerability and viral kinetics during SARS-CoV-2 human challenge in young adults. Nat Med. 2022 May;28(5):1031–41.

11. Jones BE, Brown-Augsburger PL, Corbett KS, Westendorf K, Davies J, Cujec TP, et al. The neutralizing antibody, LY-CoV555, protects against SARS-CoV-2 infection in nonhuman primates. Sci Transl Med. 2021 May 12;13(593):eabf1906.

**List of Legends**

**Table A.** Antibody concentration exponential decay rate estimated from $A_{max}$ and$A_{28}$, where $A_{28}$ is the antibody concentration at day 28. B2-7 does not have information on$A_{max}$, so the average $A_{max}$ for the other 6 participants is used instead. The values for $\alpha$ and $A_{max}$ are used in Equation (4).

**Table B.** Fitting comparison using different estimates of $\gamma$ relative to$c$.

**Table C.** Definition, units and values of model parameters. Note that the subscripts 1, 2 for each parameter refers to the sensitive and resistant virus, respectively. If a fitted parameter is shared between the two models, the population estimate for the logistic proliferation model is reported first, followed by the innate immune response model. Values with${}^{(*)}$is the average of the estimated values for the seven participants. The values for$k_{\mathrm{on}}$ reported in Table S3 are in unit of$M^{-1}s^{-1}$, while the computation is done using a conversion to$mL \mu g^{-1}\mathrm{da}y^{-1}$ (see main text).

**Fig A.** Fit of the target cell limited model with drug sensitive and resistant viral strain to the data. The circles represent viral load data, where filled green and red circles indicate viral populations dominated by either the sensitive or resistant viral strain, respectively. The unfilled circles are data below the limit of quantification or limit of detection (indicated by the two horizontal lines). Black curves are the best-fit of the model to the total viral load. The dashed–green or –red curves are model simulation of the sensitive or resistant viral strain, respectively. The first vertical red (dashed-dotted) line indicates the timing of treatment. The second vertical red (dashed) line indicates the estimated time when adaptive immunity begins to emerge. The dashed black curve shows the total viral load without treatment.

**Fig B.** Long-term viral load in the logistic and innate immune response models without adaptive immunity. Simulations are done using the best fit parameters of participant B2-8 for both models.

**Fig C.** Innate immune response model fit to data from patients who were treated with 700 mg BAM and did not rebound (part 1). The red bars indicate the limit of quantification (higher bar) and the limit of detection (lower bar).

**Fig D.** Innate immune response model fit to data from patients who were treated with 700 mg BAM and did not rebound (part 2). The red bars indicate the limit of quantification (higher bar) and the limit of detection (lower bar).

**Fig E.** Innate immune response model fit to data from control patients (part 1). The red bars indicate the limit of quantification (higher bar) and the limit of detection (lower bar).

**Fig F.** Innate immune response model fit to data from control patients (part 2). The red bars indicate the limit of quantification (higher bar) and the limit of detection (lower bar).

**Fig G.** Innate immune response model fit to data from control patients (part 3). The red bars indicate the limit of quantification (higher bar) and the limit of detection (lower bar).

**Fig H.** Distributions of $\rho$ and $\phi$ for rebound (n=7) and non-rebound (n=102) treated individuals with 700 mg BAM. Boxes in boxplots start in the first quartile and end in the third quartile of the data. The line is the median and the whiskers connect the top/bottom of the box to the max/min values that are not an outliers (data points further than 1.5 $\times$ IQR). Red crosses are outliers (outside of the whisker range).

**Fig I.** Distributions of $\rho$ and $\phi$ for treated (700 mg BAM) rebound (n=7) and placebo (n=160) individuals. Boxes in boxplots start in the first quartile and end in the third quartile of the data. The line is the median and the whiskers connect the top/bottom of the box to the max/min values that are not an outliers (data points further than 1.5 $\times$ IQR). Red crosses are outliers (outside of the whisker range).
